# miR-497-5p-RSPO2 axis inhibits cell growth and metastasis in glioblastoma

**DOI:** 10.7150/jca.62652

**Published:** 2022-01-24

**Authors:** Kun Chen, Zheng Wang, Qi-Bei Zong, Meng-Ying Zhou, Qing-Fa Chen

**Affiliations:** 1 School of Medicine, Liaocheng University, Shandong, 252000, China.; 2 Institute for Tissue Engineering and Regenerative Medicine, Liaocheng University/Liaocheng People's Hospital, Shandong, 252000, China.; 3 Department of Gastroenterology, The Second People's Hospital of Nanyang, Henan, 473000, China.; 4 Institute of Biology and Medicine, College of Life and Health Sciences, Wuhan University of Science and Technology, Hubei, 430081, China.

**Keywords:** miR-497-5p, RSPO2, Oncogenesis, The Wnt/β-catenin pathway, GBM

## Abstract

Numerous studies have found a relationship between cancer formation and aberrant microRNA expression, however the biological significance of miR-497-5p in glioblastoma (GBM) is still unknown. Compared to normal brain glial cells, miR-497-5p expression in GBM tissues was substantially lower in our study. The microRNA miR-497-5p targets R-spondin 2 (RSPO2) only when it is present. RSPO2 silencing has the same effect on GBM cells as miR-497-5p silencing, as demonstrated before. Additional mechanistic investigations have shown that miR-497-5p suppresses the Wnt/β-catenin signaling pathway by targeting RSPO2 to reduce cell proliferation, migration, and invasion. A negative correlation was discovered between MiR-497-5p and RSPO2 in 37 of the GBM tumors studied. MiR-497-5p-RSPO2 axis controls Wnt/β-catenin signaling and plays a function in GBM carcinogenesis, suggesting that it may be a therapeutic target to reduce GBM growth, as shown by our research findings.

## Introduction

A primary brain tumor called glioblastoma (GBM) has a very low survival rate 1, 2. GBM treatment is still dependent on surgical resection at this time, supplemented by radiotherapy and chemotherapy 3. Additionally neurorestorative strategies are being tried to improve its prognosis 4, 5. Due to the recurrence and metastasis of GBM patients after surgery, the results of this cancer are still unsatisfactory 6. Additionally, GBM is generated by the interplay of inherent genetic risk factors and carcinogenic environmental variables. Studies have shown that electromagnetic radiation, such as the use of mobile phones, may be related to the production of GBM 3, 6, 7. GBM has the characteristics of high proliferation and invasion, but its molecular mechanism is still unclear 8, 9. As a result, if early detection and efficient treatment are to be achieved, more research into the molecular pathways underlying the occurrence and progression of GBM is required.

Four individuals make up the R-spondin family: RSPO1, RSPO2, RSPO3 and RSPO4. These proteins are critical for embryonic development and for cancer progression 10, 11. The Wnt/-β catenin signaling pathway may be affected by RSPO2 12, 13. During mouse embryonic development, the lack of RSPO2 can cause defects in the growth of lungs, limbs, and craniofacial 14. RSPO2 has also been shown to promote EMT of gastric cancer by regulating the β-catenin signal in gastric cancer cells 13. Studies have reported that the dysregulation of RSPO2 can lead to a variety of cancers 13, 15. Nonetheless, the specific chemical mechanism through which RSPO2 is involved in GBM is unknown.

In a wide range of biological activities, an 18~24 nucleotide non-coding RNA called a microRNA controls gene expression 16. Tumor tissues have drastically changed amounts of miRNAs, according to a slew of research, indicating that these microRNAs could serve as new therapeutic targets for cancer 17-19. MicroRNA-497-5p has been identified as a miRNA that is downregulated in a number of different forms of cancer. Several oncogenes can be targeted by miR-497-5p to cause apoptosis, reduce cell proliferation, and stop cell migration and invasion in colorectal, breast, and hepatocellular carcinoma 20-22.

MiR-497-5p was studied for its role in GBM to see whether there were any mechanisms that controlled it. By directly targeting RSPO2, miR-497-5p inhibited cell proliferation *in vitro* and *in vivo*, as well as migration and invasion. Previously, it was discovered that the miR-497-5p-RSPO2 axis controls Wnt/β-catenin signaling in GBM cells.

## Materials and methods

### Tissue samples and cell lines

Liaocheng People's Hospital patients undergoing surgery who had been diagnosed with GBM had tissue samples taken from their tumors (Liaocheng, China). An ethical review board at Liaocheng People's Hospital approved the research after getting informed consent from all patients, as well as approval for clinical sample collection and usage from that hospital's medical staff. It was decided to store the samples in liquid nitrogen or formalin solution as soon as they were harvested to maximize the amount of RNA and protein available for RSPO2 analysis. The Chinese Academy of Sciences Cell Bank provided the HEB, U87, and U251 cell lines (Shanghai, China). HEB, U87, and U251 cells were grown in humidified incubators with 5% CO2 in DMEM (Hyclone, Logan, UT) supplemented with 10% FBS.

### Plasmids, siRNAs, and transfection

Lipofectamine 2000 (Invitrogen) was used to transfect cells with siRNAs or plasmids according to the manufacturer's recommendations. Gene Pharma provided siRNAs targeting RSPO2 (si-RSPO2) and non-targeting oligonucleotides (Shanghai, China). Addgene provided the human RSPO2 overexpression plasmid pcDNA3.1-RSPO2 (Cambridge, MA).

### Western blotting

The cells were lysed in RIPA buffer for 20 minutes at 0 °C. SDS-PAGE was used to isolate the proteins and transfer them to a PVDF membrane (BioRad). Primary antibodies (1:2001000) were used to incubate the membranes, followed by HRP-conjugated secondary antibodies. ECL plus reagents were used to detect protein bands (Bio-Rad). Primary antibodies used were anti-RSPO2 (17781-1-AP; Proteintech), anti-β-cantenin (8480S; Cell Signaling Technology), anti-phosphorylated β-catenin (Ser33/37/Thr41) (#9561, Cell Signaling Technology), anti-CyclinD1 (ab16663; Abcam) and anti-GAPDH (2188; Cell Signaling Technology).

### Cell proliferation assay

A Cell Titer 96 Aqueous One Solution (MTS) kit, as directed by the manufacturer, was utilized to assess cell proliferation (Promega).

### Immunohistochemistry

Assays for immunohistochemistry were carried out as previously described 22, 23. In brief, the sample is dewaxed, rehydrated and then antigen repaired. The sections were then incubated with the primary antibody overnight. Anti-Ki67 (27309-1-AP; Proteintech) and anti-RSPO2 (17781-1-AP; Proteintech) were utilized as primary antibodies. The sections were then incubated with the secondary antibody for one hour at room temperature. Finally, DAB developer was added for dyeing. RSPO2 protein expression was scored according to the percentage of positive cells and staining intensity. Percentage scores were 1 (<25%), 2 (25-50%), 3 (51-75%), and 4 (76-100%). The staining intensity was divided into 0 (no staining), 1 (week), 2 (medium) and 3 (strong). The final score was calculated as a multiple of staining percentage and staining intensity.

### Wound-healing assay

Using a pipette tip, a horizontal scratch was made on the monolayer after the cells had been stabilized overnight on a 6-well plate. After being gently washed twice with PBS, the cells were grown in a serum-free medium and examined under a microscope for 24, 48, and 72 hours.

### *In vitro* migration and invasion assays

As previously mentioned, migratory and invasion experiments were done 23, 5 × 10^4^ cells were used for migration and invasion.

### Real-time RT-PCR

The TRIzol reagent was used to obtain total RNA from cells and tumor tissues (TaKaRa, Japan). MLV reverse transcriptase (Invitrogen) was used to turn 2 mg of total RNA into cDNA, and each cDNA sample was analyzed in triplicate using SYBR Green (Tiangen, China) according to the manufacturer's instructions. The following were the primer sequences used in the experiment: RSPO2 (human), 5'- ACAATACTGTGTCCAACCAT-3' and 5'- TCCTCTTCTCCTTCGCCTTT-3' and GAPDH (human), 5'- ATGACATCAAGAAGGTGGTG -3' and 5'- CATACCAGGAAATGAGCTTG -3'.

### Animal studies

4-week-old BALB/c-nu (nude) mice were obtained from SLAC Laboratory Animal Co., Ltd (Shanghai, China). 1×10^7^ Lines of GBM cells. The rear flanks of mice were injected with stably overexpressing miR-497-5p or control groups. Afterward, the mice were euthanized 35 days later after being photographed. The tumor volume and weight are determined by carefully removing the tumor tissue from the genital area. All animals were cared for and treated in compliance with the criteria established by Liaocheng University's Animal Ethics Committee.

### Statistical analysis

Each experiment's result is calculated using the mean standard deviation of at least three separate studies. SPSS v13.0 was used to analyze the test data. The statistical description of the experimental data in each group was represented by x±S. An ANOVA with Tukey correction and a t-test were employed to see whether there was a difference between the groups. * P < 0.05. ** P < 0.01.

## Results

### RSPO2 is significantly upregulated in human GBM

**Figure 1:**
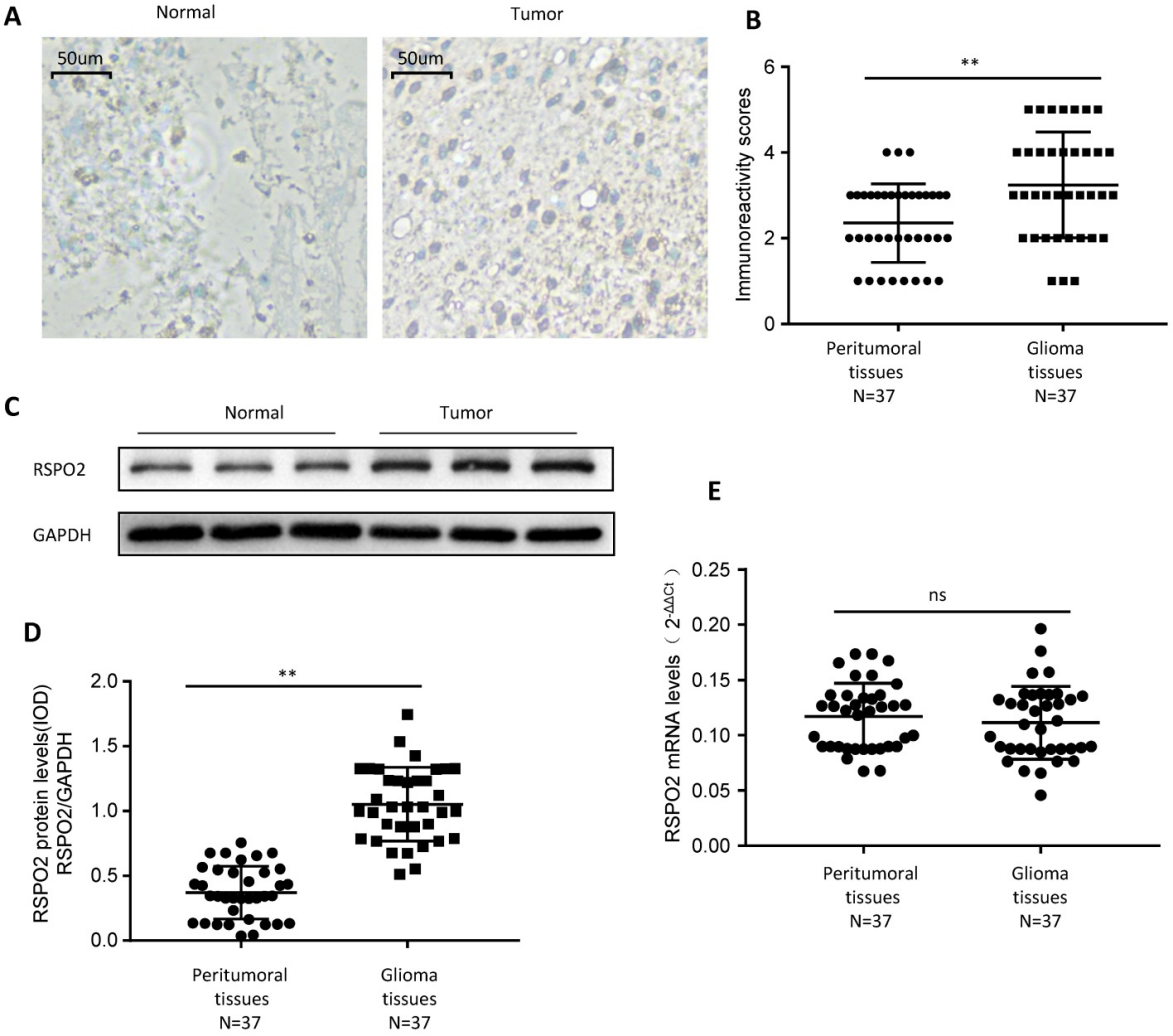
** RSPO2 is upregulated in human glioblastoma. (A, B)** Immunohistochemistry of RSPO2 expression in 37 glioblastoma tissues and peritumoral tissues. Representative immunohistochemistry images (A) and semi-quantitative evaluation (B) of RSPO2 protein expression. **(C-E)** Analysis of RSPO2 expression in 37 glioblastoma tissues and peritumoral tissues. Representative western blotting images of RSPO2 protein levels in three glioblastoma tissues and three peritumoral tissues (C). RSPO2 and GAPDH protein levels were determined via densitometry using ImageJ and are represented as IOD (D). RSPO2 mRNA levels were determined by real-time RT-PCR (E). Data represent the means ± SEM. **P < 0.01. ns, not significant.

First, we looked at whether RSPO2 expression was elevated in paraffin-embedded GBM tissues and peritumoral tissues from 37 people to see if it was. GBM tissues displayed higher levels of RSPO2 expression than peritumoral tissues, according to immunochemistry (Figure 1A-B). We discovered that RSPO2 protein levels were elevated, but that RSPO2 mRNA levels were not using frozen tissue for protein and mRNA extraction (Figure 1C-E). These data imply that GBM tissues as a whole have greater levels of RSPO2 expression.

### RSPO2 promotes GBM cells proliferation, migration and invasion

**Figure 2:**
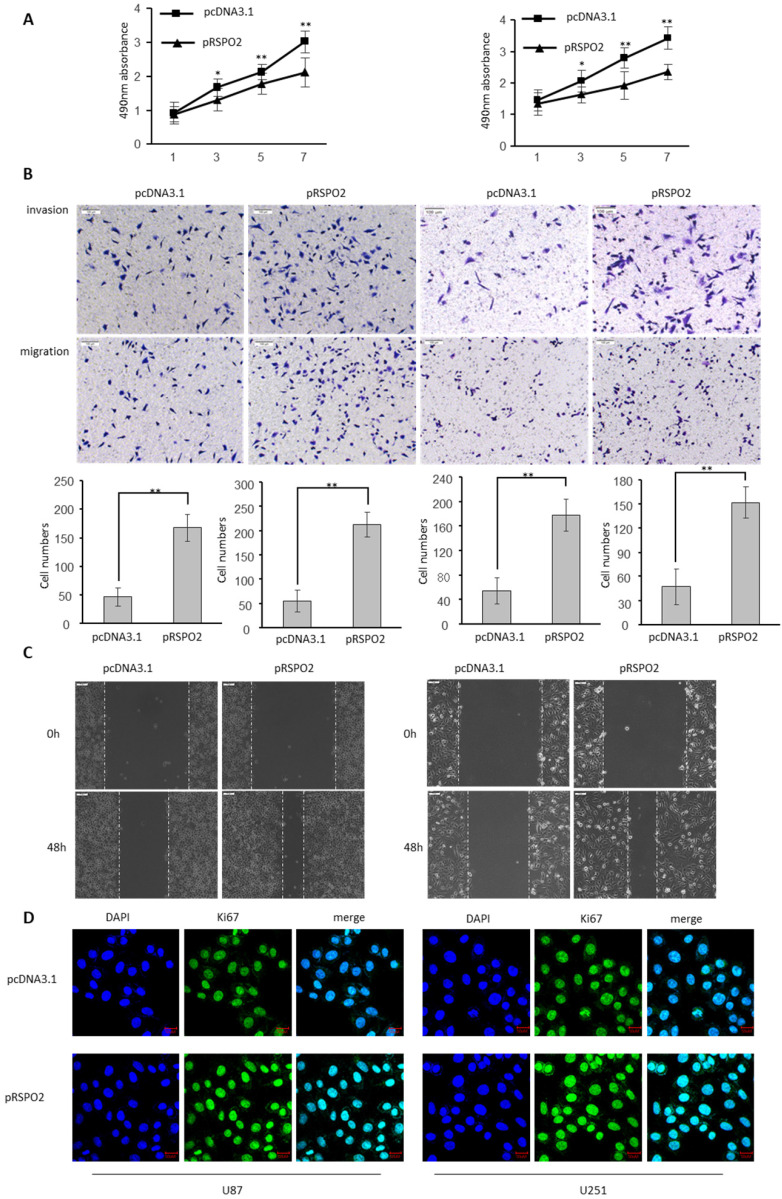
** RSPO2 promotes GBM proliferation, migration and invasion.** U87 and U251 cells were transduced with RSPO2 expression plasmid (pRSPO2) or pcDNA3.1 as indicated. **(A)** Cell proliferation was determined at the indicated time points by MTS assay. **(B,C)** Cell metastasis was determined by Transwell assays (B) and Scratch wound assays. **(D)** Immunofluorescence was used to detect the changes of the proliferation gene Ki67. Data represent the means ± SEM. **P < 0.01.

The biological significance of RSPO2 in GBM was investigated by transfecting U87 and U251 cells with either an RSPO2-overexpressing or a control plasmid (pRSPO2) (pcDNA3.1). When cell viability in GBM cells was assessed using the MTS test, overexpression of RSPO2 significantly improved cell vitality when compared to the control group (Figure 2A). It is clear from Figure 2B how much more rapidly the RSPO2 overexpression group multiplied and migrated than the control group. Overexpression of RSPO2 in a wound-healing experiment boosted cell migration, which is in line with this. Increased cell migration was seen during wound healing *in vitro* when RSPO2 was overexpressed (Figure 2C). RSPO2 dramatically boosted GBM cell proliferation as measured by the expression of the cell proliferation marker Ki67 (Figure 2D). As expected, the cell proliferation invasive and migratory ability, and the expression of KI67 were significantly elevated when transfected with siRSPO2 (Figure S1).

### RSPO2 is a direct target of miR-497-5p

**Figure 3:**
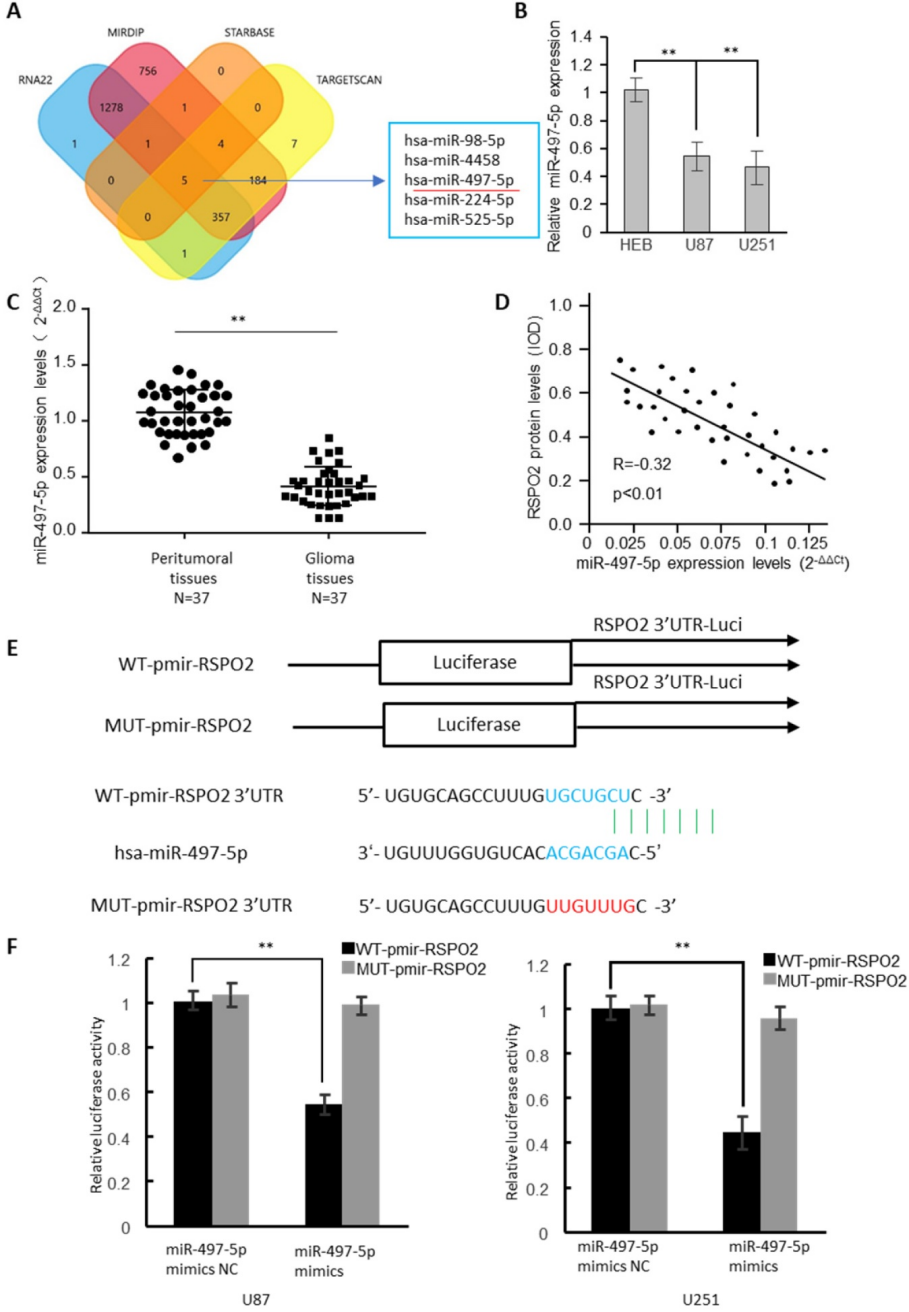
** RSPO2 is a direct target of miR-497-5p. (A)** The four-way Venn diagram reveals the numbers of overlapping miRNAs obtained using four publicly available bioinformatics algorithms and the microarray-based RSPO2 signature. **(B)** Real-time RT-PCR was used to detect the relative expression of miR-497-5p in normal glial cells and glioblastoma cells. **(C)** Analysis of miR-497-5p expression in 37 glioblastoma tissues and peritumoral tissues. **(D)** Correlation between miR-497-5p levels and RSPO2 levels in 37 glioblastoma tissues. **(E)** Nucleotide predicted miR-497-5p-binding site in the RSPO2 mRNA 3′-UTR. **(E)** Luciferase activities were measured in U87 and U251 cells transfected with miR-497-5p mimics or miR-497-5p mimics NC and WT-pmir-RSPO2 or MUT-pmir-RSPO2. Data represent the means ± SEM. **P < 0.01.

In the RSPO2 3'-UTR, four bioinformatics approaches identified the presence of five potential miRNA binding sites (Figure 3A). MiRNA-497-5p expression was dramatically reduced in GBM cells, as determined by real-time RT-PCR analysis (Figure 3B). 37 GBM tumors and neighboring normal tissues were found to have significantly reduced expression of miR-497-5p by identifying (Figure 3C). The RSPO2 protein and miR-497-5p were found to be antagonistic in human GBM tissues, as well (Figure 3D). There have been two reporter vectors designed to test whether or not this predicted binding site regulates specifically; one contains the coding sequence for Luciferase followed by either the wild type or mutant RSPO2 3′-UTR (Figure 3E). Mice transfected with Wt-RSPO2 and miR-497-5p had lower luciferase activity, while mice transfected with Mut-RSPO2 had higher luciferase activity (Figure 3F). There is some evidence to suggest that RSPO2 is capable of directly binding to miR-497-5p.

### miR-497-5p inhibits migratory and invasive abilities of GBM cells

**Figure 4:**
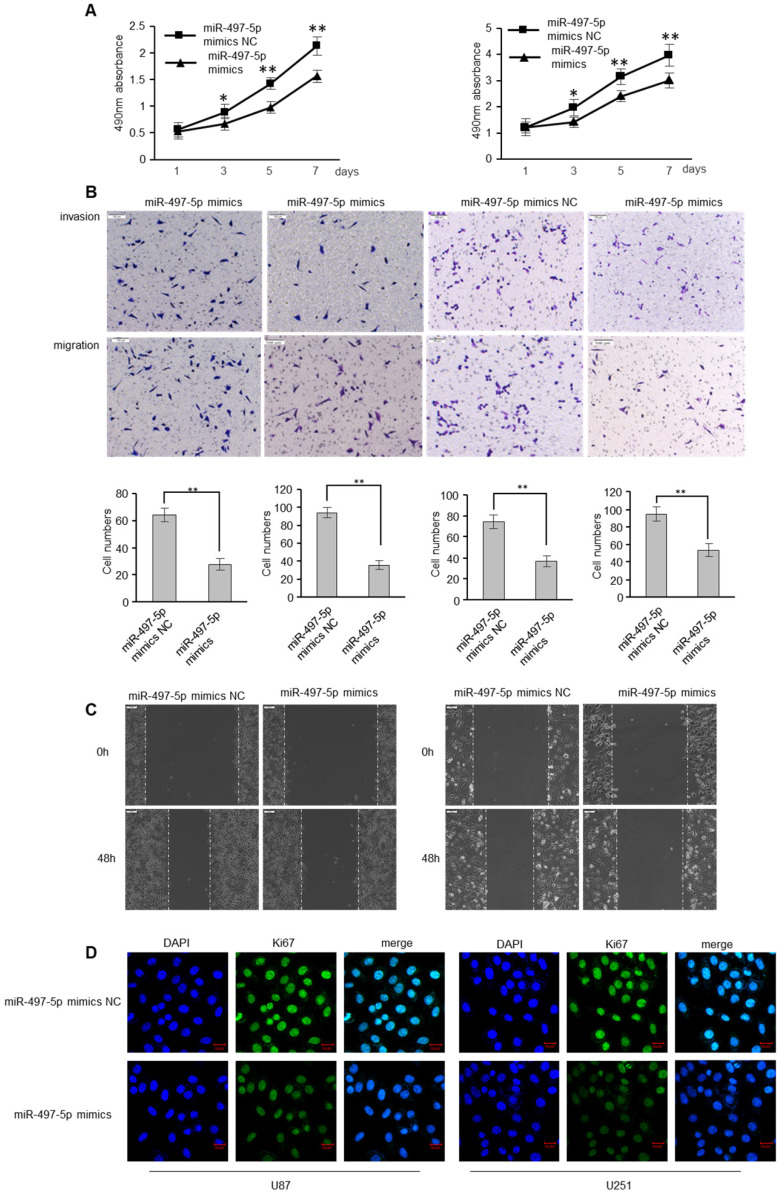
** miR-497-5p inhibits migratory and invasive ability of GBM cells.** U87 and U251 cells were transduced with miRNA-497-5p mimic NC or miRNA-497-5p mimics. **(A)** MTS assay indicted that miR-497-5p mimics inhibited ability of proliferation. **(B)** Chamber invision ability was damaged in miRNA-497-5p mimics cells. **(C)** Cell would healling ability was impaired in miRNA-497-5p mimics cells. **(D)** The expression levels of Ki67 were detected by immunofluorescence. Data represent the means ± SEM. **P < 0.01.

To determine whether miR-497-5p has an influence on GBM cell apoptosis, GBM cells were transfected with miR-497-5p mimics or mimic controls. As expected, miR-497-5p mimics inhibited GBM cell proliferation, as determined by the MTS experiment (Figure 4A). MiR-497-5p mimics also decreased GBM cell motility and invasion, which was consistent with the effects of RSPO2 overexpression (Figure 4B-C). Comparing the experimental and control groups, immunofluorescence analysis also demonstrated decreased Ki67 expression (Figure 4D). As expected, transfecting cells with a miR-497-5p inhibitor increased cell proliferation, migration, and invasion (Figure S2).

**Figure 5:**
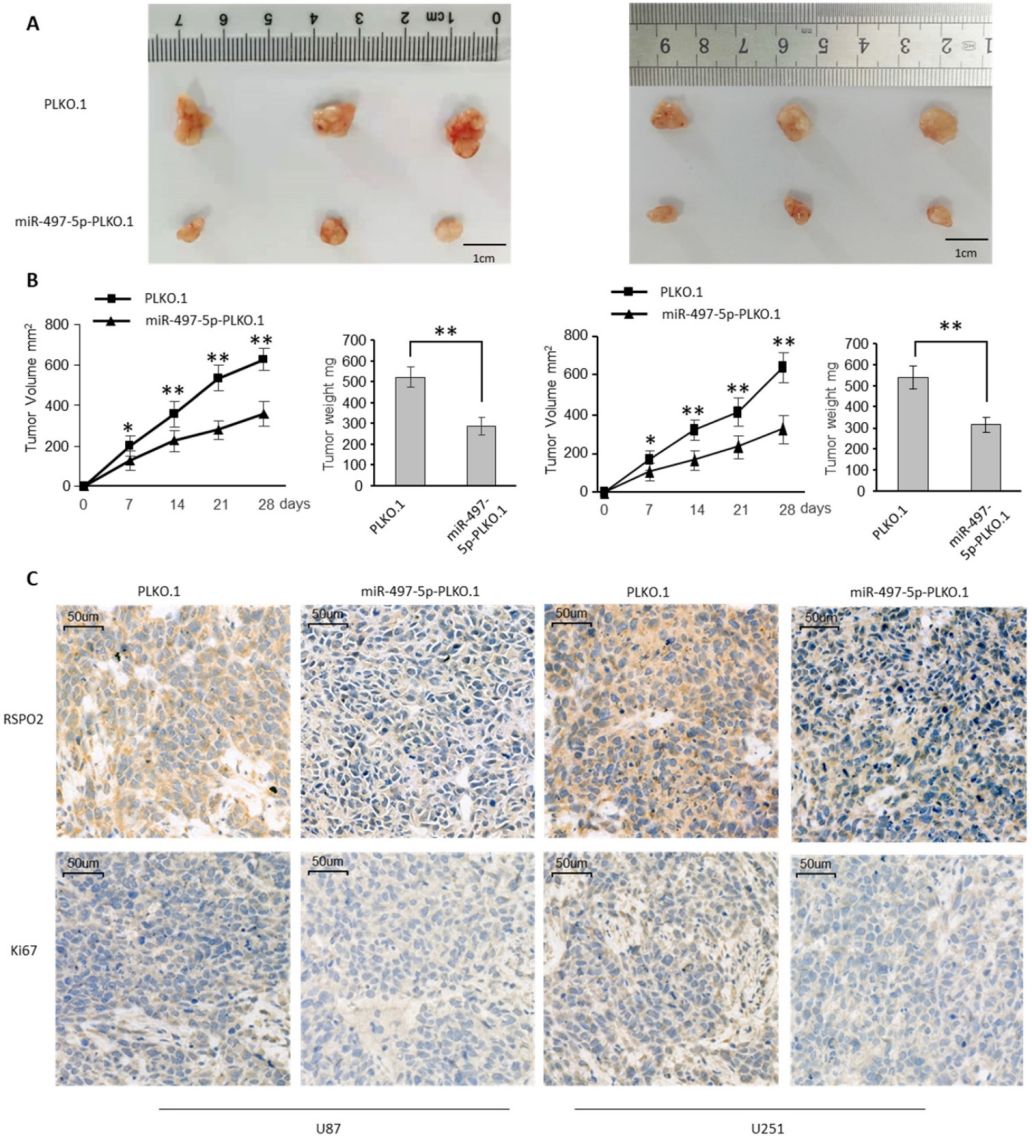
** miR-497-5p suppresses glioblastoma growth *in vivo*.** Subcutaneous xenografts of GBM cells infected with miR-497-5p overexpressing lentivirus (plko.1-miR-497-5p) or control lentivirus (plko.1). **(A)** Images of the tumors at autopsy from nude mice are presented. **(B)** Tumor volumes and average weight of xenografted tumors were measured. **(C)** Immunohistochemical (IHC) staining of RSPO2 and Ki67 in xenografted tumors from plko.1-miR-497-5p cells or control cells. Data represent the means ± SEM. **P < 0.01.

To investigate the impact on GBM metastasis, we created two GBM cell lines that were stably overexpressing miR-497-5p (miR-497-5p- plko.1). MiR-497-5p - plko.1 is a non-coding RNA. The -plko microRNA (miR-497). Intraperitoneal injections of either a test cell line or a control cell line were administered to nave mice (plko.1). Thirty days following the inoculation, the mice were slaughtered and the miR-497-5p-plko.1 groups' average tumor volume and weight were greatly reduced1 (Figure 5A-B). Tumor tissue from nude mice from the experimental group displayed lower levels of Ki67 and RSPO2 expression than that from the control group, according to immunohistochemistry (Figure 5C). As a result of this study, we may conclude that miR-497-5p can inhibit cancer cell growth *in vivo*.

### miR-497-5p-RSPO2 axis regulates Wnt/β-catenin signaling in GBM cells

**Figure 6:**
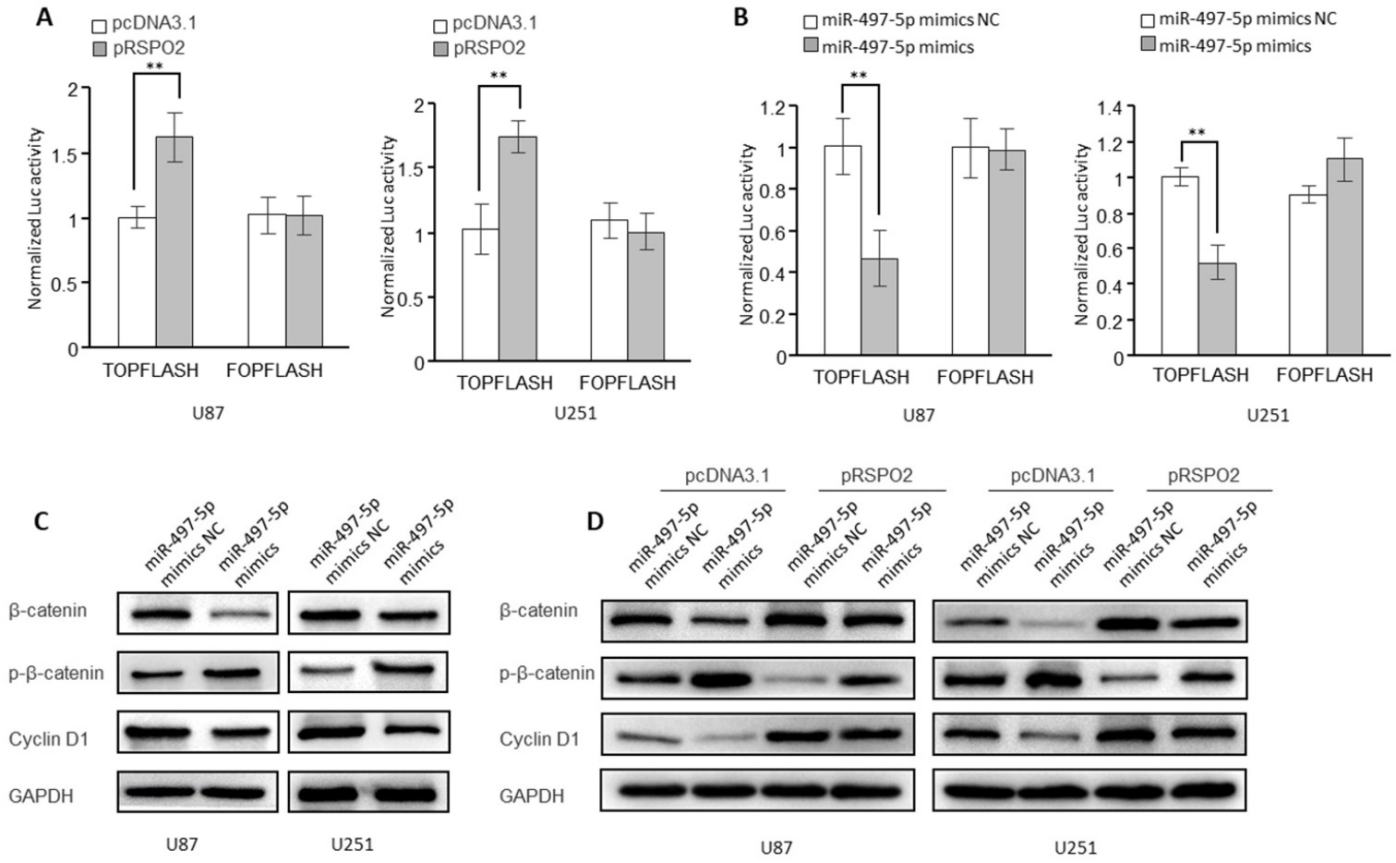
** miR-497-5p-RSPO2 axis regulates Wnt/β-catenin signaling in GBM cells. (A, B)** β-catenin reporter assay in U87 and U251 cells with RSPO2 overexpression (A) or miR-497-5p overexpression (B). **(C)** Effects of miR-497-5p on protein levels of total β-catenin, phosphorylated β-catenin (Ser33/37/Thr41), cyclin D1. **(D)** RSPO2 partially restored the levels of total β-catenin, phosphorylated β-catenin (Ser33/37/Thr41), cyclin D1. Data represent the means ± SEM. **P < 0.01.

According to research, RSPO2 has been linked to cancer by the Wnt/β-catenin signaling pathway and may be carcinogenic in some cases. It was for this reason that we looked into whether or if the receptor for the transcription factor RSPO2 is also involved in GBM. Indeed, in GBM cells, overexpression of the RSPO2 gene increased the activity of the β-catenin reporter (TopFlash) (Figure 6A). However, miR-497-5p had the opposite effect on the activity of the β-catenin reporter (Figure 6B). When miR-497-5p was overexpressed in GBM cells, the phosphorylated forms of β-catenin (Ser33/37/Thr41) rose, whereas the total expression of β-catenin decreased. As a result, the cyclin D1 (a Wnt target gene) was considerably suppressed (Figure 6C). In addition, miR-497-5p-induced increases in β-catenin total, as well as phosphorylated and cyclin D1 levels, were partially reversed by RSPO2 overexpression (Figure 6D). Wnt/β-catenin signaling in GBM cells was inhibited by miR-497-5p together via targeting RSPO2.

## Discussion

MiR-497-5p expression was observed to be lower in GBM tissues compared to peritumoral tissues in this research. When miR-497-5p was irregularly introduced, cell growth, migration, and invasion were all reduced *in vitro*. In clinical sample analysis, MiR-497-5p and RSPO2 had a negative correlation. While this study focuses on glioblastomas, previous studies have shown that miR-497-5p serves as a tumor suppressor gene in a number of cancers, including leukemia and lymphoma 24-28. According to these findings, lower levels of miR-497-5p are linked to both the occurrence and progression of cancer.

As one of the most common types of brain cancer, glioblastoma has no reliable early diagnosis or treatment methods 29, 30. At the moment, GBM is mostly treated with a combination of surgical resection, radiation, and chemotherapy. While surgery can efficiently remove the tumor, it will result in significant trauma and a prolonged healing period 29, 30. Thus, researching the molecular pathogenesis of glioblastoma and devising new targeted therapies have become the focus of contemporary research. MicroRNA has been widely investigated in GBM, and deregulation of miRNA can result in carcinogenesis. Because of this, it's possible that abnormal miR-497-5p expression in GBM is linked to an increased risk of developing the disease.

RSPO2 is a member of the RSPO protein family, which includes other members such as RSPO1. RSPO2's role in cancer is unclear; it could be an oncogene or a tumor suppressor. Previous studies show that RSPO2 can enhance the growth of gastric cancer and hepatic carcinoma but inhibits colorectal cancer 13, 31, 32. Cell proliferation, migration, and invasion were all decreased when either RSPO2 or miR-497-5p was silenced, as we discovered. In order to prevent the Wnt pathway from working, scientists used β-catenin signals.

**Figure 7:**
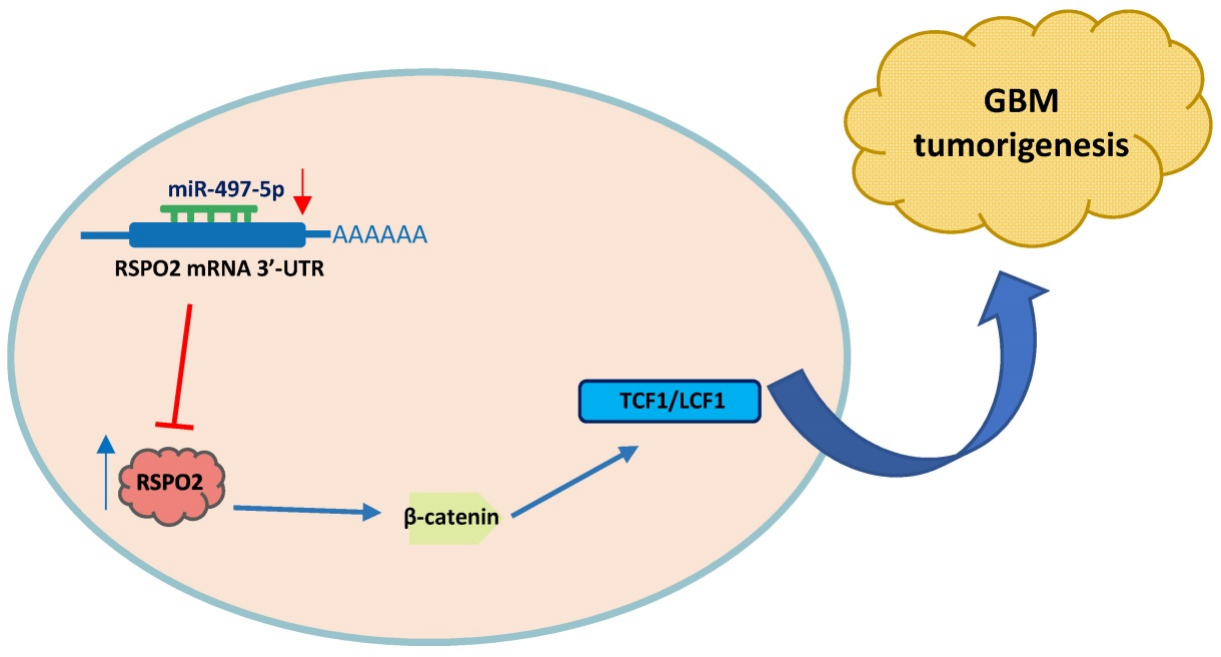
** Schematic diagram of roles of miR-497-5p-RSPO2 axis on Wnt/β-catenin signaling and its function in glioblastoma tumorigenesis.** In GBM, there is reduced levels of miR-497-5p leading to elevated RSPO2 expression and activation of the Wnt/β-catenin signaling pathway.

In summary, miR-497-5p regulates the expression of RSPO2 in GBM cells and tissues, according to the findings. RSPO2 and the resulting phosphorylation of both β-catenin and total β-catenin by MiR-497-5p restrict GBM cell growth and metastasis, as seen in Figure 7. As we learn more about how GBM develops, we expect that MiR-497-5p will serve as both a predictive biomarker and a therapeutic target.

## Supplementary Material

Supplementary figures.

## Abbreviations

IOD: integrated optical density
UTR: untranslated region
GBM: Glioblastoma

## References

[1] Ostrom QT, Gittleman H, Truitt G, Boscia A, Kruchko C, Barnholtz-Sloan JS (2018). CBTRUS Statistical Report: Primary Brain and Other Central Nervous System Tumors Diagnosed in the United States in 2011-2015. Neuro Oncol.

[2] Wen PY, Reardon DA (2016). Neuro-oncology in 2015: Progress in glioma diagnosis, classification and treatment. Nat Rev Neurol.

[3] De Boeck A, Ahn B, D'Mello C, Lun X, Menon S, Alshehri M (2020). Glioma-derived IL-33 orchestrates an inflammatory brain tumor microenvironment that accelerates glioma progression. Nature communications.

[4] Review of clinical nerve repair strategies for neurorestoration of central nervous system tumor damage Journal of Neurorestoratology. 2020; 8: 172-81.

[5] Impact of preoperative Karnofsky Performance Scale (KPS) and American Society of Anesthesiologists (ASA) scores on perioperative complications in patients with recurrent glioma undergoing repeated operation Journal of Neurorestoratology. 2019; 7: 143-52.

[6] Cheng M, Zhang ZW, Ji XH, Xu Y, Bian E, Zhao B (2020). Super-enhancers: A new frontier for glioma treatment. Biochim Biophys Acta Rev Cancer.

[7] Bastola S, Pavlyukov M, Yamashita D, Ghosh S, Cho H, Kagaya N (2020). Glioma-initiating cells at tumor edge gain signals from tumor core cells to promote their malignancy. Nature communications.

[8] Wang J, Su HK, Zhao HF, Chen ZP, To SS (2015). Progress in the application of molecular biomarkers in gliomas. Biochem Biophys Res Commun.

[9] Cargnelutti E, Ius T, Skrap M, Tomasino B (2020). What do we know about pre- and postoperative plasticity in patients with glioma? A review of neuroimaging and intraoperative mapping studies. Neuroimage Clin.

[10] Bayard Q, Nault J, Zucman-Rossi J (2020). RSPO2 abnormal transcripts result from read-through in liver tumours with high ß-catenin activation and mutations. Gut.

[11] Seshagiri S, Stawiski EW, Durinck S, Modrusan Z, Storm EE, Conboy CB (2012). Recurrent R-spondin fusions in colon cancer. Nature.

[12] Knight M, Karuppaiah K, Lowe M, Mohanty S, Zondervan R, Bell S (2018). R-spondin-2 is a Wnt agonist that regulates osteoblast activity and bone mass. Bone research.

[13] Zhang H, Han X, Wei B, Fang J, Hou X, Lan T (2019). RSPO2 enhances cell invasion and migration via the WNT/β-catenin pathway in human gastric cancer. J Cell Biochem.

[14] Reis A, Sokol S (2021). Rspo2 inhibits TCF3 phosphorylation to antagonize Wnt signaling during vertebrate anteroposterior axis specification. Scientific reports.

[15] Longerich T, Endris V, Neumann O, Rempel E, Kirchner M, Abadi Z (2019). RSPO2 gene rearrangement: a powerful driver of β-catenin activation in liver tumours. Gut.

[16] Loyer X, Paradis V, Hénique C, Vion AC, Colnot N, Guerin CL (2016). Liver microRNA-21 is overexpressed in non-alcoholic steatohepatitis and contributes to the disease in experimental models by inhibiting PPARα expression. Gut.

[17] Bandiera S, Pfeffer S, Baumert TF, Zeisel MB (2015). miR-122-a key factor and therapeutic target in liver disease. J Hepatol.

[18] Goodall GJ, Wickramasinghe VO RNA in cancer. 2021; 21: 22-36.

[19] Sun X, Jiao X, Pestell T, Fan C, Qin S, Mirabelli E (2014). MicroRNAs and cancer stem cells: the sword and the shield. Oncogene.

[20] Wang H, Yu M, Hu W, Chen X, Luo Y, Lin X (2019). Linc00662 Promotes Tumorigenesis and Progression by Regulating miR-497-5p/AVL9 Axis in Colorectal Cancer. Front Genet.

[21] Li X, Wang Q, Rui Y, Zhang C, Wang W, Gu J (2019). HOXC13-AS promotes breast cancer cell growth through regulating miR-497-5p/PTEN axis. Journal of cellular physiology.

[22] Zhang M, Yan X, Wen P, Bai W, Zhang Q (2021). CircANKRD52 Promotes the Tumorigenesis of Hepatocellular Carcinoma by Sponging miR-497-5p and Upregulating BIRC5 Expression. Cell transplantation.

[23] Zhang HM, Li H, Wang GX, Wang J, Xiang Y, Huang Y (2020). MKL1/miR-5100/CAAP1 loop regulates autophagy and apoptosis in gastric cancer cells. Neoplasia.

[24] Li G, Wang K, Wang J, Qin S, Sun X, Ren H (2019). miR-497-5p inhibits tumor cell growth and invasion by targeting SOX5 in non-small-cell lung cancer. Journal of cellular biochemistry.

[25] Gharib E, Nasri Nasrabadi P, Reza Zali M (2020). miR-497-5p mediates starvation-induced death in colon cancer cells by targeting acyl-CoA synthetase-5 and modulation of lipid metabolism. Journal of cellular physiology.

[26] Cheng L, Xing Z, Zhang P, Xu W (2020). Long non-coding RNA LINC00662 promotes proliferation and migration of breast cancer cells via regulating the miR-497-5p/EglN2 axis. Acta Biochim Pol.

[27] Omuro A, DeAngelis L (2013). Glioblastoma and other malignant gliomas: a clinical review. JAMA.

[28] Fridrichova I, Kalinkova L, Karhanek M, Smolkova B, Machalekova K, Wachsmannova L (2020). miR-497-5p Decreased Expression Associated with High-Risk Endometrial Cancer. International journal of molecular sciences.

[29] Wu W, Yu T, Wu Y, Tian W, Zhang J, Wang Y (2019). The miR155HG/miR-185/ANXA2 loop contributes to glioblastoma growth and progression. J Exp Clin Cancer Res.

[30] Ahir BK, Ozer H, Engelhard HH, Lakka SS (2017). MicroRNAs in glioblastoma pathogenesis and therapy: A comprehensive review. Crit Rev Oncol Hematol.

[31] Yin X, Yi H, Wang L, Wu W, Wu X, Yu L (2017). R-spondin 2 promotes proliferation and migration via the Wnt/β-catenin pathway in human hepatocellular carcinoma. Oncol Lett.

[32] Dong X, Liao W, Zhang L, Tu X, Hu J, Chen T (2017). RSPO2 suppresses colorectal cancer metastasis by counteracting the Wnt5a/Fzd7-driven noncanonical Wnt pathway. Cancer Lett.

