# Supplementary material for: miR-497-5p-RSPO2 axis inhibits cell growth and metastasis in glioblastoma
Source: J Cancer. 2022 Jan 24;13(4):1241–51. doi: 10.7150/jca.62652 (PMC8899375; doi:10.7150/jca.62652)
Supplement: Supplementary file 1 — Supplementary figures. [file jcav13p1241s1.pdf]

Supplementary Figure 1. Silencing RSPO2 reduces proliferation, migration, and invasion of GBM cells.

U87 and U251 cells were transfected with siRSPO2 or siCon. (A) Cell proliferation was determined by MTS assay. (B, C) Cell metastasis was determined by Transwell assays (B) or Scratch wound assays (C). (D)The expression levels of the cell proliferation marker Ki67 were detected by immunofluorescence. Data represent the means ± SEM. **P < 0.01.

Supplementary Figure 2. miR-497-5p inhibitor promotes migratory and invasive ability of GBM cells.

U87 and U251 cells were transduced with miRNA-497-5p inhibitor NC or miRNA-497-5p inhibitor. (A) MTS assay indicted that miRNA-497-5p inhibitor promoted ability of proliferation. (B) Chamber invision ability was elevated in miRNA-497-5p minics cells. (C) Cell would healling ability was improved in miRNA-497-5p inhibitor cells. (D) The expression levels of Ki67 were enhanced by immunofluorescence. Data represent the means ± SEM. **P < 0.01.

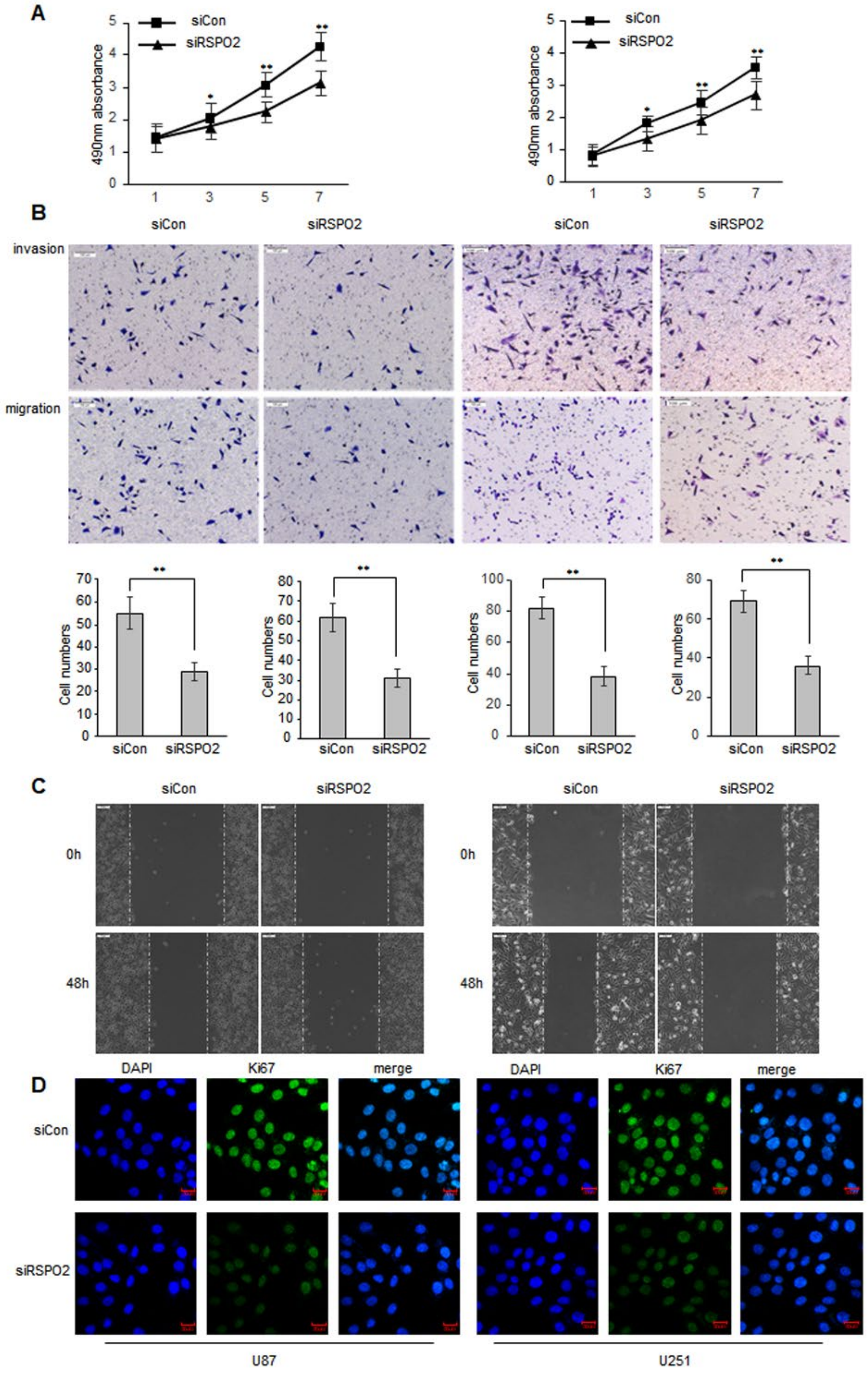

A
siCon
siRSPO2
490nm absorbance
B
siCon
siRSPO2
invasion
migration
Cell numbers
C
0h
48h
D
DAPI
Ki67
merge
U87
U251

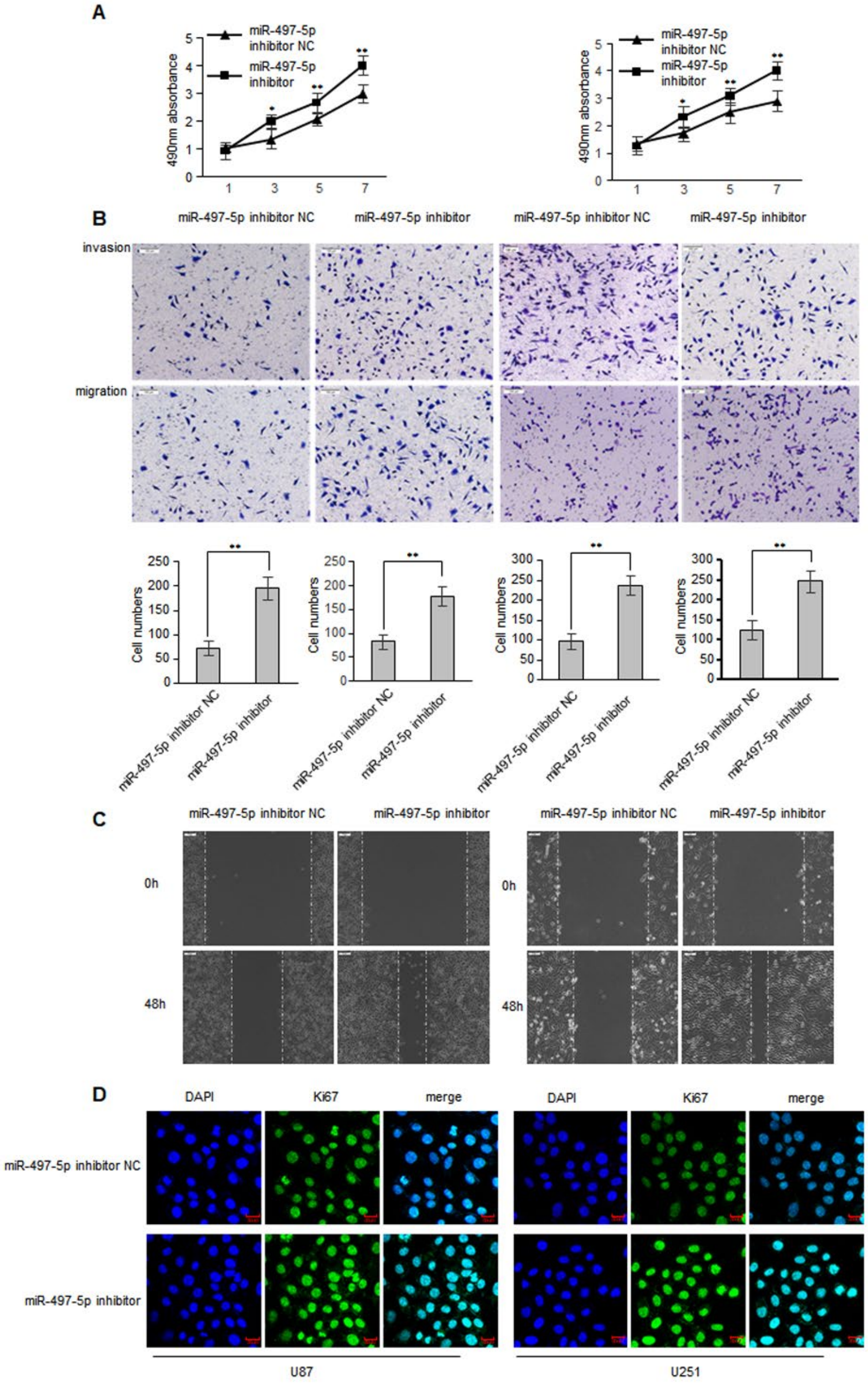
A
miR-497-5p inhibitor NC
miR-497-5p inhibitor
490nm absorbance
B
miR-497-5p inhibitor NC
miR-497-5p inhibitor
invasion
migration
Cell numbers
C
0h
48h
D
DAPI
Ki67
merge
miR-497-5p inhibitor NC
miR-497-5p inhibitor
U87
U251
